# Adapting a digital intervention to prevent youth violence and depressive symptoms from the emergency department for community violence interventions

**DOI:** 10.1080/28324765.2025.2600722

**Published:** 2025-12-12

**Authors:** Lauren A. Magee, Jennifer Leaño, Beatrice Beverly, Katrina Nelson, Guangyu Tong, Megan Ranney

**Affiliations:** a Children’s Health Services Research, Department of Pediatrics, Indiana University School of Medicine, Indianapolis, IN, USA; b Yale School of Public Health, Yale University, New Haven, CT, USA; c Genesys Solutions, Indianapolis, IN, USA; d Yale School of Medicine, Yale University, New Haven, CT, USA

**Keywords:** Firearm injury, firearm violence exposure, digital-based intervention, community violence intervention, credible messenger

## Abstract

Firearm injury is the leading cause of death among youth in the US direct and indirect exposure is associated with increased mental health needs, particularly depression, yet few community-based interventions led by credible messengers exist to address co-occurring violence and depression among youth. This paper describes a pilot study to adapt a digital intervention for youth exposed to firearm violence. iDOVE3.0 was adapted from an evidence-based emergency department intervention for youth (ages 13–17) into a community setting (defined as a community-based organization outside an institutional setting) in Indianapolis, Indiana. This single-arm pilot study aimed to recruit 20 youth between September 1, 2024 and December 31, 2025. Participant recruitment is ongoing, and to-date we have screened 16 youth for mild to moderate depression and violence exposure and enrolled five youth. Violence patterns and depressive symptoms were assessed at baseline and follow-up at 2, 4 and 8 months. This descriptive study offers insights into the adaptation of a clinical intervention and implementation process into a community setting. Understanding how digital-based interventions can expand community violence interventions and how credible messengers can improve the acceptability of digital interventions are promising approaches to address co-occurring depression and violence among youth in need. Future studies will examine feasibility, acceptability and preliminary efficacy among the pilot study cohort.

## Introduction

Firearm injury is the leading cause of death among youth in the United States (Lee et al., 2022; Roberts et al., 2023). In 2022, there were 6.7 youth firearm-related deaths per 100,000 population (Villarreal et al., 2024), with nearly twice as many nonfatal injuries (Kaufman et al., 2020). In addition to death and injuries, youth are indirectly exposed to firearm violence through peers, families, and community violence (Magee et al., 2022; Martin et al., 2022; Semenza et al., 2023). For instance, in a nationwide sample of young people, nearly one-quarter of young people witnessed violence in the past year (Finkelhor et al., 2015). Furthermore, in cities like Chicago nearly one-third of youth have been indirectly exposed to firearm injury, meaning that they heard gunshots and/or knew someone who was shot (Lennon et al., 2024). Direct and indirect exposure to firearm violence has adverse health and mental health outcomes – particularly depression (Lennon et al., 2024; Magee et al., 2022, 2023). Youth exposed to violence (victim/family) experience depressive symptoms at three times the rate of community peers and higher rates of gun ownership in adulthood (Caves Sivaraman et al., 2023; Magee, Aalsma, 2022; Magee et al., 2022; Ranney et al., 2019). The cycle of exposure leading to future injury is driven by prior violence exposure, involvement in violence (Wamser-Nanney et al., 2019), and long-term disparities in social and economic outcomes (Jay et al., 2019; Mehranbod et al., 2022; Oliphant et al., 2019; Wong et al., 2020).

Youth identify the need for emotional and mental health support following direct and/or indirect exposure to firearm violence (Borthwell et al., 2021; Magee et al., 2023; Oddo et al., 2021, 2024). However, engagement rates with mental health providers following a firearm injury remain low (Magee et al., 2022; Oddo et al., 2024; Ranney et al., 2020). Furthermore, systematically identifying youth exposed to firearm violence and prospectively connecting youth with interventions are in their infancy. Clinical screening tools, such as saFETy (serious fighting, friend weapon-carrying, community environment and firearm treats) were developed to assess firearm victimization and firearm exposure in adult emergency departments (ED) (Goldstick et al., 2017). A recent study in the ED in three cities demonstrated that the saFETy screening tool was effective in identifying firearm violence exposure among young adults (18–24 years), but the connection to appropriate interventions remains unknown (Goldstick, Carter, 2024; Goldstick et al., 2024). When the saFETy tool was implemented in a pediatric primary care setting in Chicago and referred youth in need of community organizations, it failed to identify youth (12–17 years) at the highest exposure risk (Goyal et al., 2024). Taken together, these studies successfully demonstrate that screening tools integrated in clinical settings can connect youth with needed care (Goyal et al., 2024); however, in order to reach youth at elevated risk and with less access to care, interventions need to connect with credible messengers and engage with community groups outside a clinical setting.

Emerging research has demonstrated the importance of engagement with trusted community groups and credible messengers in addressing concurrent depressive symptoms and violence exposure (Lesnick et al., 2023; Magee et al., 2023; Szkola & Blount-Hill, 2025). Credible messengers lead Community Violence Interventions (CVIs), which are promising for mentorship, for improving conflict management, for providing mental health support, and some evidence indicates reductions in future involvement in violence (Buggs et al., 2022; Hureau & Papachristos, 2024; Ross et al., 2023; Webster et al., 2012, 2013). Given the established avenue for trusted connections within communities, integrating violence assessment tools into CVI’s may be a promising avenue. The CVI workforce, however, is insufficient, and resources within community groups are often limited to serve many young people (Community Violence Intervention Action Plan, 2024; Mongelli et al., 2020). Digital interventions are scalable solutions and have demonstrated a reduction in peer violence and depression symptoms among adolescents in pediatric EDs (Ranney et al., 2016; Ranney et al., 2018, 2019). However, firearm violence screening and digital interventions within a community setting to reduce depression and violence among youth are understudied. New research is needed to translate digital interventions into broadly accessible and sustainable community-based interventions to reduce depressive symptoms and prevent future violence for vulnerable youth.

In this study, we adapted and piloted a text-message intervention previously used in the ‘Intervention for Depression and Violence Prevention in the ED (iDOVE)’ a bundle strategy targeting cognitive, emotional, and self-efficacy pathways that commonly lead to violence and depression. A recent randomized controlled trial of iDOVE in the emergency department demonstrated significant intervention effects among youth with higher levels of violence and moderate depression at baseline and eight-week follow-up, with extremely high acceptability, feasibility, retention, and satisfaction (Ranney et al., 2016, 2018).

We hypothesized that iDOVE would be acceptable and feasible within a mid-sized community setting that experiences higher rates of firearm violence than the national average (Villarreal et al., 2024) and would help extend existing CVI services. In this study, we describe the rationale and the process of adapting an ED intervention into a community-setting, partnering with a community-based mental health provider and CVI group, and piloting iDOVE.

## Methods

### Intervention overview

The purpose of this pilot single-arm study is to adapt an evidence-based ED intervention for youth (ages 13–17) into a community setting (defined as a community-based organization outside an institutional setting) in Indianapolis, Indiana. The target enrollment is 20 youth between September 2024 and December 2025. iDOVE was developed, refined, and piloted by an emergency physician and violence prevention researcher (MR) for a pediatric emergency department (Ranney et al., 2018, 2019). iDOVE3.0 is a two-part intervention that consists of (1) a 15–20 min scripted brief intervention (BI) based on motivational interviewing (MI) and cognitive behavioral therapy (CBT) delivered by a trained bachelor level research assistant, and (2) eight weeks of interactive, tailored, theoretically informed text messages (‘Text’) (Ranney et al., 2016, 2019). The overall goals of the intervention modifications were to increase the number of youth with the greatest need within communities that experience high rates of firearm violence (Magee et al., 2022; Villarreal et al., 2024), improve intervention efficacy, and ultimately reduce violence and depressive symptoms among youth in need.

The iDOVE3.0 intervention and informed consent process was approved by the Indiana University Institutional Review Board (#21700).

### Setting and community partnership

Indianapolis, Indiana, is a midwestern city of just under 1 million population. Annual rates of firearm violence are 65.5 per 100,000 (Magee et al., 2022), 48 per 100,000 youth population (Magee, 2024), and ranks 23^rd^ in the nation for youth at risk for depression (Silverman). Compared to a national average of 11%, 15% of Indianapolis residents live below the federal poverty line (Bureau USC, 2024).

Through previously established trusted community-engaged partnerships (Damaris et al., 2024; Magee et al., 2023), two community-based organizations focused on mental health and violence prevention serve as the community connection. Both are CVIs that primarily serve Black and Brown communities and offer youth mentoring, life coaching, violence intervention, child mental health services and family support through a trauma-informed care model. Both organizations are deeply rooted in community mental health and violence prevention coalitions and provide services in many charter-based high schools. These avenues allow for multiple opportunities to recruit from a diverse pool of potentially eligible youth. The CBO received two training sessions from the ED iDOVE research team on the cognitive-based therapy brief intervention and the text-based intervention. The training sessions included watching the ED and the text interventionist conducting the iDOVE intervention, followed by active role plays. Both CBO organizations were compensated for their participation and time through a stipend from the university.

### Intervention adaption

The adaptation of the iDOVE ED-based intervention into a community setting was guided by the Framework for Reporting Adaptations and Modifications-Expanded (FRAME) (Wiltsey Stirman et al., 2019). Modifications to the original iDOVE were made prior to implementation. The decision to modify the intervention from the ED into the community (i.e. iDOVE3.0) was informed by prior community-based research (Magee et al., 2022, 2023) and field-based work within the community (Magee, 2024). Modifications were made to the content and contextual aspects of the intervention. The content was adapted to include measures of violence exposure (i.e. how many gunshots have you heard?), in addition to prior measures of physical violence as a victim and/or perpetrator in the screening, baseline, and follow-up surveys. This modification was made because of the known adverse mental health outcomes from violence exposure among youth (Magee et al., 2022). The contextual modifications were made in the setting and personnel – from the ED to the community setting for study recruitment and brief intervention delivery. The brief intervention was delivered by a community-based mental health provider, who is a trusted source and community leader (i.e. credible messenger) among youth, instead of a research assistant (Lesnick et al., 2023).

### Recruitment

iDOVE3.0 opened for enrollment on September 1, 2024, and is ongoing for participant recruitment with a target goal of 20 youth. Recruitment of youth occurred through multiple mechanisms: (1) Parents and youth are invited to information sessions by both CVIs. (2) A study flyer was shared with other local community groups with basic information about the study and study team contact information for interested youth and/or caregivers to inquire about eligibility. (3) In-person recruitment occurs through brief one-on-one information sessions with youth currently working with a community-based mental health provider and/or through community-based organizations word of mouth. All recruitment meetings took place in a community setting (i.e. library, community group headquarters, participant residence, etc.).

Youth took a brief screening survey through the Research Electronic Data Capture (REDCap) System (Harris et al., 2009, 2019) (approximately 5 min) to ensure he/she/they meet the inclusion criteria for enrollment. Youth were eligible for screening if: (1) 13–17 years old; (2) English speaking; (3) had access to a cell phone; and (4) the ability to obtain parent or guardian consent. The brief screen occurred at the time of recruitment within a community setting or at a scheduled time following parent/caregiver verbal consent with the CVI mental health provider.

### Eligibility

Youth were eligible for enrollment into iDOVE3.0 if they self-reported (1) past-year peer (non-partner/family) physical violence (victimization or perpetration) or indirect exposure to firearm violence, using a modified version of the SaFETy (serious fighting, friend weapon carrying, community environment, and firearm threats) score, a validated measure that screens for the frequency and variety of physical victimization and perpetration, as well as family and community violence exposure (Goldstick et al., 2017). (2) Mild-to-moderate depressive symptoms, defined as a score of 5–19 on the Patient Health Questionnaire-9 (PHQ-9), a standard (Allgaier et al., 2012; Kroenke et al., 2001; Moriarty et al., 2015), well-validated screening tool for depressive symptoms; and (3) ownership or access to a text-message-capable mobile phone. For youth who met the eligibility criteria, they would complete a second written consent/assent and a detailed contact information form.

### Intervention procedures

Once youth meet screening criteria and enrolled in iDOVE3.0, youth receive (1) a brief intervention that last 15–30 min and (2) daily, tailored text messages for eight weeks. Enrolled participants receive a BI that includes concepts of CBT and MI-based intervention, which includes discussion on the thoughts-feelings-actions triangle, encouraging goal setting and reflection regarding self-efficacy on violence exposure and depressive symptoms. The BI is delivered by a community mental health provider (e.g. a credible messenger) within a community setting (e.g. CVI headquarters). At the time of enrollment, participants are also oriented to the daily text message, and youth are able to choose what time of day the daily mood query text is received (outside of school hours), use their own phone, and choose how they name iDOVE in their phone. SMS text-messages are tailored by high violence and low violence, based on the baseline violence exposure level.

Text content consists of (1) Automated daily mood queries (‘Hi, this is iDOVE3.0. How are you feeling today? (1 = really bad, 5 = great, never better)’); (2) Automated daily messages, tailored according to daily mood score and baseline violence levels or exposure; and (3) On-demand supportive messages, which can be automatically pulled by the participant texing key words ‘stressed’, ‘sad’, ‘angry’ or ‘happy’. Such texts aims to develop cognitive reappraisal, emotional regulation, and self-efficacy skills over the 8-week intervention (‘When you feel upset or angry, try writing things down to get your thoughts out of your head. Many teens say this helps. (No one has to see what you write!)’).

### Outcome measures and data collection

Once enrolled in the study, all participants take surveys at baseline and follow-up at 2, 4, and 8 months to assess violence patterns, depressive symptoms, and potential covariates. The primary outcome of violence exposure is measured using the same modified version of the SaFETy score (score = 0: low violence, 1–5: medium violence, ≥6: high violence) as that used in the screening (Goldstick et al., 2017, 2024). Questions were modified to identify broader violence patterns, such as ‘how often have you heard gun shots?’ and ‘how many family members or friends have been hurt by violence?’ (Goyal et al., 2024) Depressive symptoms were assessed using the Center for Epidemiologic Studies Depression Scale Revised [CESD-R] (score = 1–4: minimum depression, 4–9 mild, 10–14 moderate, 15–19 moderate severe, ≥ 20: severe depression), which captures a wider range of depressive symptoms in addition to the PHQ-9 which was given during screening (Eaton et al., 2001).

To assess potential covariates, the presence of post-traumatic stress symptoms are measured using the Primary Care PTSD scale (score ≥ 3 = positive PTSD), violence-related self-efficacy using the Bosworth Violence Self-Efficacy Scale ( Dahlberg et al., 2005) (total score 5 = less confidence; 25 = very confident) and identify firearm behavior patterns using the Firearm carriage and access survey (FIRE) at baseline and follow-up. Socio-demographic variables such as race, ethnicity, family poverty and academic performance are assessed using measures from the National Study of Adolescent Health (Harris & Udry, 2018), with additional questions on gender identity from the Gender Identity in the US Surveillance (GenIUSS) group ( Reisner et al., 2015) are assessed at baseline.

At the two-month follow-up, we will conduct a semi-structured qualitative interview to assess the perception of the in-person/text-based topics, text-based program structure, length, frequency, and overall strengths and weaknesses of iDOVE3.0. We will also ask participants who they recommend delivering the intervention and who they recommend participating on an advisory panel. The participants will take two surveys to assess the user experience with the BI and the text-messaging intervention. Likert-scale survey questions are grounded in seven constructs of the Theory of Acceptability to assess how appropriate participants deem the intervention (Sekhon et al., 2017). Example questions include asking participants about the likability of iDOVE3.0, how appropriate the length was, and if they thought iDOVE3.0 had a positive effect on them. These assessments will help determine acceptability and help refine the intervention for future community-based iDOVE studies.

### Protection of participants

There are several mental health safeguards in place to ensure the safety of any youth who may experience a crisis. Crisis protocols include responding to youth within 24 h, contacting parents if youth are unresponsive, and calling 911 if needed. All youth who reported low mood scores and/or are unresponsive were contacted by the community mental health provider. All the text responses are reviewed daily by a research team member. Any responses deviating from the expected parameters (e.g. a youth sends unexpected words, such as ‘help’ or ‘hurt’) will automatically receive an automated text message instructing them to call the community groups, a 24/7 mental health hotline (i.e. 988) or 911 in case of emergency. The system also reminds the youth that the system is not monitored by a person in real time, and a research team member will follow up with the youth and/or caregiver if needed.

In alignment with prior work on the iDOVE in the ED setting, participants received a maximum compensation of $215 for the entire trial ($20 for baseline, $50 for 2-month follow-up, including qualitative interview, $60 for 4-month follow-up, and $65 for 8-month follow-up. $20 for the cell phone plan for participants). The amount for each follow-up covers the youth’s time to conduct the intervention and offers compensation for the use of their phone. Youth received Visa gift cards in person.

### Analysis

After study recruitment has been completed, we use both quantitative and qualitative measures to determine feasibility and acceptability. To determine feasibility, we calculate completion rates, the average time of completion for assessment surveys, and follow-up rates for daily text message responses. We will assess differences in baseline characteristics and changes in depressive symptoms and violence using chi-squared and t-tests with 95% confidence intervals. If data are missing due to participant drop out, we will censor the data at the point of loss.

All qualitative interviews will be audio recorded, transcribed, and stored by the participant ID number in NVivo on a password protected server. We plan to conduct deductive (based on prior iDOVE studies) and inductive thematic coding analysis. The coding structure was iteratively refined, a codebook was developed, and each interview was coded by three independent reviewers. Reviewers discuss all discrepancies until a consensus is reached. The most common themes and subthemes will be independently interpreted and discussed with the whole research team.

## Results

iDOVE3.0 opened for enrollment on September 1, 2024, and is ongoing for participant recruitment with a target goal of 20 youth. Our study team regularly holds meetings to discuss recruitment and presents the intervention to parents/caregivers and youth through community presentations and the community mental health provider. To date, 16 youth have taken the screening survey, and 13 have completed the screening. Of those who completed the survey, 9 identified as Black, 3 identified as White, and 1 identified as American Indiana/Native; 10 identified as female and 4 identified as male. Most were 17 years of age (*n* = 5), in 11^th^ and 12^th^ grade (*n* = 7), and 79% qualified for free or reduced lunch.

Four respondents were deemed ineligible for iDOVE3.0; three youth had low/no depression (scores 0, 3, 4), and one youth did not have access to a cell phone. All the respondents were screened for medium and high levels of violence exposure. Of the 9 individuals deemed eligible for iDOVE3.0, two were screened for mild depression (scores 5, 6), five for moderate depression (scores 11, 12 [2], 13 [2]) and two for moderately severe depression (scores 16, 19). Five were screened for medium risk of violence (scores of 3, 4, 5 [3]), and four were screened for high risk of violence (scores 6, 7, 9, 13). Of the 9 eligible participants for iDOVE, five have successfully enrolled in the on-going intervention. The remaining four youth plan to enroll but not because of scheduling conflicts, family emergencies, and lack of transportation to the community headquarters. We are actively trying to enroll them and recruiting more youth into the study.

At baseline, two youth were screened for moderate depression (scores 13, 14), two for moderate severe depression (scores 17, 18), and one for severe depression (score 39); four youth were screened for medium risk for violence (scores 4, 3, 3 & 5), and one for high risk for violence (score 8). Youth scored in the lower (scores 10, 11) and middle range (16, 3, 3) on their ability to control their anger and resolve conflicts. Three youth screened positive for possible posttraumatic stress disorder (scores = 3). Two youth indicated that it would be easy to obtain a firearm in their neighborhood, but none of the youth indicated that they carried a firearm in the past year.

To date, 91% (range, 18–59) of youth have responded to daily text messages. One youth requested an on-demand message (angry) and the youth preferred text messages between 3pm and 6pm (mean 4:40pm).

## Discussion

iDOVE3.0 is a digital-based intervention aimed to reduce depressive symptoms and violence among youth and is a novel addition to existing CVIs. Several aspects of our study are innovative and have important implications on preventive digital-based interventions within community-based CVI organizations, which are still in their infancy. First, we adapted an evidence-based ED text-based program into the CVI program of violence reduction with the goal of reaching more youth in need of behavioral health support who are at an elevated risk of being involved in violence. Second, a credible messenger trained in providing brief CBT-based interventions delivered the brief intervention and led recruitment efforts. A crucial piece of CVI work is the credible messenger, a person with lived experience and one that a youth can better identify with culturally and socially.

Finally, we expanded the inclusion criteria for iDOVE3.0 to include youth who have been indirectly exposed to firearm violence beyond direct victimization and/or offending. A growing body of research demonstrates that youth exposed to violence are often at the highest risk for being involved in violence and experiencing greater mental health needs. We used the saFETy screening tool (Goldstick et al., 2017, 2024), which was previously only administered in clinical settings; however, a growing body of research demonstrates that indirect firearm violence exposure through the community and family increases mental health needs among youth (Leibbrand et al., 2020, 2021; Lennon et al., 2024; Magee et al., 2022). For example, youth exposed to a traumatic event are 2.6 times greater odds of receiving a diagnosis of depression than non-exposed youth (Vibhakar et al., 2019), and depressive symptoms among youth increases the risk of involvement in future violence (Borowsky & Ireland, 2004; Cuevas et al., 2010; Magee et al., 2022; Ranney et al., 2013). iDOVE3.0 focuses on prospective ascertainment of youth who have been exposed to violence and who are experiencing on depressive symptoms without the need to present for care in a clinical setting. Nine of the youth screened as medium and high risk for violence and moderate depression, indicating that we are recruiting the appropriate youth population.

To date, we have described the adaptation of a clinical intervention into a community CVI setting and the preliminary feasibility of recruiting and enrolling youth in the iDOVE3.0 intervention within a community setting. CVI practitioners typically provide street outreach, violence interruption, life coaching, hospital-based violence intervention, and violence-focused cognitive behavioral therapy. This work has largely been focused on the period immediately following a shooting event to prevent retaliatory violence. iDOVE3.0 aims to expand the scope of CVI programs, and by the end of the pilot study, we hope to demonstrate the acceptability of iDOVE with the use of a credible messenger, further refine the intervention based on participant feedback, and determine the preliminary efficacy of iDOVE3.0 on depressive symptoms and exposure to violence. The process of refining the intervention with feedback from credible messengers and youth allows for the intervention to be adapted to different populations and communities. For instance, consistent with prior iDOVE studies, we focused on youth depression, yet we are also assessing probable PTSD at baseline screening to explore the prevalence of PTSD symptoms among a cohort of violence-exposed youth. Prior studies indicate a high prevalence of PTSD among similar populations (Goldstick et al., 2024; Magee et al., 2022). Future versions of the intervention may be adapted to address PTSD depending on the findings.

We are also recruiting through one community health and CVI organization, but future versions of the intervention could potentially allow for broader screening of youth exposed to violence through other evidence-based CVI programs within the community. Therefore, extending the reach of behavioral health support through iDOVE3.0 and CVI credible messengers to engage youth with the greatest need but are often missed in other programs.

The initial phase of this pilot study has not been without its challenges. Enrolling eligible youth into the intervention has been slower than anticipated because of scheduling conflicts (e.g. sports practices, school events) on weekends, family emergencies to include the death of a loved one, and a lack of transportation to community headquarters to participate in brief intervention. Our team is continuously reviewing protocols and trying to reduce such barriers when possible. For instance, providing transportation vouchers can be provided to the community headquarters, as in prior studies (Richardson et al., 2021). Future work will also aim to study the intervention through an implementation science framework to assess the barriers and facilitators to implementation.

To our knowledge, this descriptive pilot study is the first to adapt an ED-based digital intervention into community health and CVI work. This study has the ability to inform how to adapt and implement an evidence-based ED digital health intervention in CVI programing to address both violence reduction and youth mental health. This would be an important step forward for the expansion and sustainability of CVI behavioral health work. Future work will include qualitative interviews with both CVI staffers and CBO leadership to further assess the implementation of iDOVE and identify opportunities to further refine the intervention. Lastly, findings from this study will also provide scientifically important information and inform future randomized control trials and/or tailoring and adapting the intervention to improve implementation.

## Data Availability

The data that support these findings of study are available from the corresponding author (LM), upon reasonable request.
